# Impacts of immersive 3D videos on students’ surgical learning compared to 2D videos: a randomized controlled trial

**DOI:** 10.1097/JS9.0000000000002146

**Published:** 2024-11-25

**Authors:** Amelia Favier, Eya Jaafar, Raphael L’Hote, Philippe Gauthier, Ignacio Avellino, Geoffroy Canlorbe

**Affiliations:** a Department of Gynecological and Breast Surgery and Oncology, Pitié-Salpêtrière, Assistance Publique des Hôpitaux de Paris (AP-HP), University Hospital, Paris, France; b Sorbonne Université, Inserm, UMR 938, Centre de Recherche Saint Antoine, Equipe Instabilité des Microsatellites et Cancer, Paris, France; c Sorbonne Université, CNRS, INSERM, ISIR, Institut des Systèmes Intelligents et de Robotique, Paris, France; d Sorbonne Université, Faculté de médecine, AP-HP, Groupement Hospitalier Pitié Salpêtrière, Centre National de Référence du Lupus Systémique, du syndrome des antiphospholipides et autres maladies auto-immunes, Service de Médecine Interne 2, Institut E3M, CIMI-Paris, Paris, France; e Sorbonne University, Centre de Recherche Saint-Antoine (CRSA), INSERM UMR_S_938, Cancer Biology and Therapeutics, Paris, France; f University Institute of Cancer, Sorbonne University, Paris, France; g BOpA, Bloc Opératoire Augmenté, AP-HP, l’Institut Mines-Télécom et l’Université Paris-Saclay

**Keywords:** 3D, head-mounted display, immersive video, medical education, surgical learning

## Abstract

**Background:**

Unlike other medical specialties, surgery is primarily learned through apprenticeship, by observing surgeons in action during operations. However, the increasing number of medical students and work-hour restrictions limit opportunities for learning in the operating room (OR). These circumstances call for novel technologies, such as immersive video. The objective of this study is to compare knowledge retention, preparedness, and content engagement for surgical learning when watching a surgical video in 3D through a Head-Mounted Display (HMD) versus in 2D video on a conventional screen.

**Materiel and Methods:**

This randomized controlled trial includes 231 fourth-year medical students. Participants watched the same 12 min surgical video, narrated by an expert, presented either in immersive 3D form through an HMD, or in 2D form through a conventional screen. The students completed three questionnaires (previewing, postviewing, and 1 month later), which included questions on knowledge retention, expected preparedness, content engagement, tool engagement, and self-assessment. Statistical analyses were adjusted by including the confounding factors.

**Results:**

Immediately after the video, the 3D immersive video group showed a significantly lower knowledge retention score compared to the classic video group (*P*<0.01). Nonetheless, the 3D immersive video group demonstrated better-expected preparedness as a future resident (*P*=0.019), greater satisfaction (*P*=0.033), better stimulation (*P*<0.001), higher involvement (*P*<0.001), and a greater perceived ability to identify anatomical structures (*P*<0.001). After 1 month, participants in the immersive video group reported feeling more prepared (*P*=0.016), more self-confident (*P*=0.020), more at ease (*P*=0.023), and less overwhelmed (*P*<0.01) than those in the 2D video group.

**Conclusion:**

Our results showed that 3D surgical immersive video vs. 2D surgical video, enhances the sense of stimulation, satisfaction, involvement, and the perception of having better identified anatomical structures. For early medical school students where access to the OR is limited, this tool appears to a significant step forward in surgical pedagogy. However, the precise understanding of its pedagogical value required further investigation and refinement.

## Introduction

Highlights

- Surgery: Learning to perform surgery is a long endeavor that occurs not only in lecture halls, but also largely through practice in the operating room.
- Immersive video: Immersive video overcomes problems of access to the operating room.
- Objective: to compare knowledge retention, preparedness, and content engagement for surgical learning when watching a surgical video in 3D through a head-mounted display (HMD) versus in 2D video on a conventional screen.

Learning to perform surgery is a long endeavor that occurs not only in lecture halls, but also largely through practice in the operating room (OR). During internships in various surgical departments, students acquire knowledge through observation and, gradually, by assisting surgeons under their supervision. However, several factors currently limit surgical practice, including the increasing number of medical students, work-hour restrictions, and financial or ethical concerns related to animal and cadaveric models^1–4^. These circumstances highlight the need for novel technologies that allow students to learn, practice, and perfect their skills in preparation for performing procedures on patients. Among the systems used for surgical education, virtual reality (VR) takes center stage. For example, students can develop manual skills using VR simulation systems, as studies have shown they are a viable alternative to physical trainers^5^. Theoretical knowledge can also be acquired in VR, as visualizing anatomy in 3D has been shown to enhance learning compared to 2D, particularly when using immersive means to visualize 3D models with a head-mounted display (HMD)^6,7^. The development of technical skills is particularly compelling in VR, as students can engage in interactive simulations to repeat training exercises, such as executing gestures, monitoring their performance, increasing training difficulty, and working on team management, all without putting patients at risk^6,8–11^. Moreover, the rapid influx of HMD devices into the market has allowed students to experience VR immersion, marking a significant advancement in learning, especially in the field of medicine^12^. However, creating virtual environments with 3D interactive objects incurs high costs due to the significant amount of time required to develop content. In contrast to VR simulations, video material can be produced more easily, as surgeons can summarize technical gestures and the relationships between structures in video recordings, thus enhancing students’ understanding of a procedure compared to traditional training methods and significantly improving the acquisition of surgical skills^13,14^. A recent meta-analysis indicated that comparing 2D videos viewed on screens to 3D videos experienced in immersion not only measured knowledge retention but also assessed ancillary factors such as confidence, engagement, distraction, and interest. The findings demonstrate that immersive videos create an environment conducive to effective learning, fostering deeper comprehension and retention^15^. Many of these advantages arise from the fact that students visualize real surgeries rather than simulations. The realistic presentation enhances medical students’ understanding of the progression of clinical procedures, effectively preparing them for their upcoming participation in the OR^16^. Given the benefits of realistic materials and their relatively low production costs, it is pertinent to study the effects of replacing virtual environments with video in immersive learning systems. However, the few studies that have measured actual learning outcomes provide limited evidence. It remains unclear how adding immersion to video impacts learning outcomes^17^. Our hypothesis is that immersive 3D surgical videos vs. 2D surgical videos, improves knowledge retention in early medical students, as well as expected preparedness and preparedness after 1 month.

## Materials and methods

### Participants

**Figure 1:**
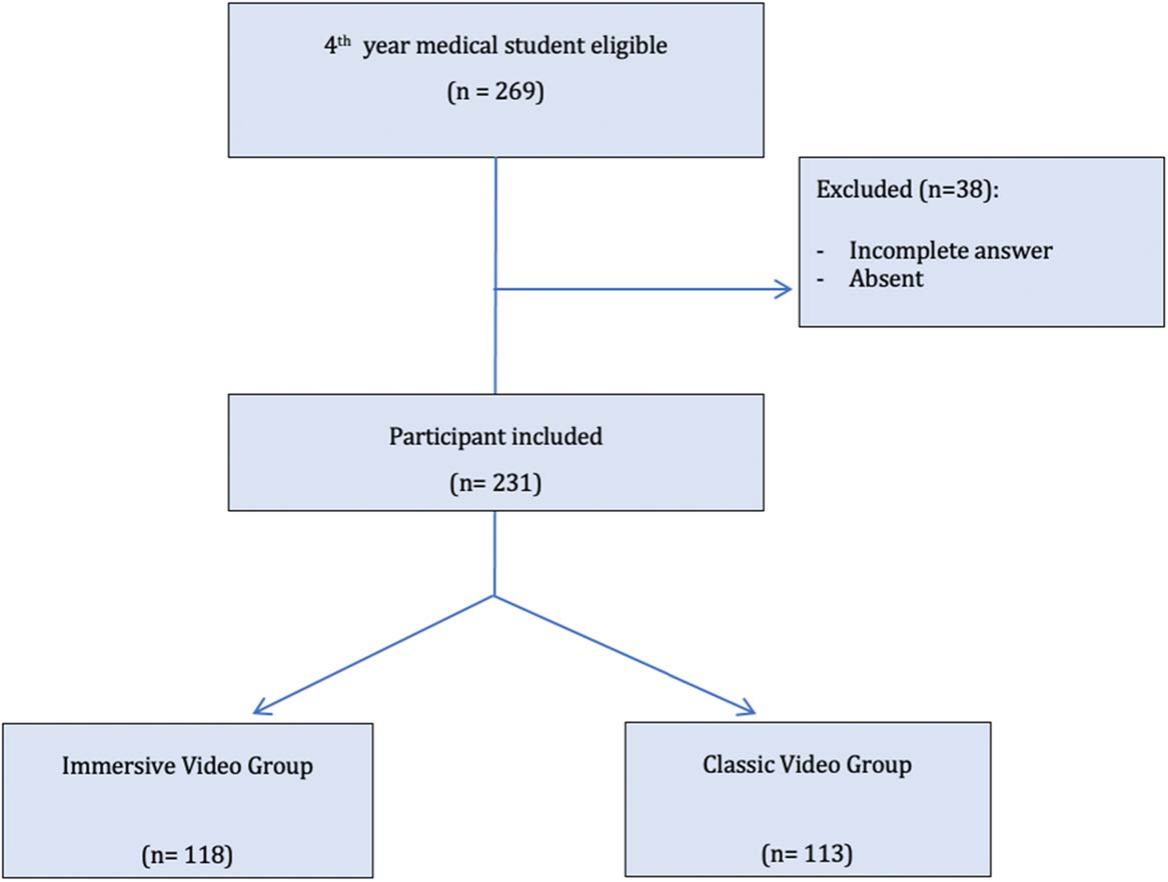
CONSORT flow diagram.

Inclusion criteria were all fourth-year medical students at a university who were in the simulation course (Fig. 1). This teaching was mandatory and consists of 4 h classes for each student. The course was divided into three sessions of 5 days each (December 2022, March 2023, and May 2023).

Exclusion criteria were students who did not want to participate, or who have repeated a year.

### Objectives

The objective of this study was to compare knowledge retention, preparedness, and content engagement for surgical learning when watching a 3D surgical immersive video through a head-mounted display (HMD) versus a 2D video on a conventional screen.

### Experimental design

From the start of the simulation class, students were randomly assigned (1:1) using R software (blockrand function), with no difference between participants, to one of the two groups: the 3D immersive surgical video (HMD) or the 2D surgical video (laptop computer). There were no difference in demographics criteria between the two groups. On arrival, they were notified of their anonymous number and their group.

The students watched the same 12 min surgical video, narrated by an expert, presented either in immersive 3D form through a HMD, or in 2D form through a conventional screen. The video showed a real C-section that has been recorded using a Z-Cam K1 3E camera preserving the patient’s anonymity. The video showed three members of the obstetrics and gynecology team during the C-section: an expert operator performing the procedure, assisted by a primary (resident) and secondary (early medical student) assistant. Viewers could see each members gestures as if their eyes were a meter away from the surgical site inside the OR. The recording was edited to create a 12 min lecture, divided into chapters, including introduction, positioning, cutaneous incision, digitoclasia, uterine incision, caudal extraction, placental delivery, uterine closure, hemostasis, aponeurosis closure, and cutaneous closure. As the lecture plays, a studio-recorded voice of an expert in obstetrics and gynecology narrates the content, explaining the gestures, techniques, and the anatomy of the main uterine vessels, as well as the purpose of the instruments used at each step, prohibited gestures, and the reasons they are dangerous. The lecture included essential knowledge to understand a classic C-section procedure, along with the role of each surgical team member.

**Figure 2:**
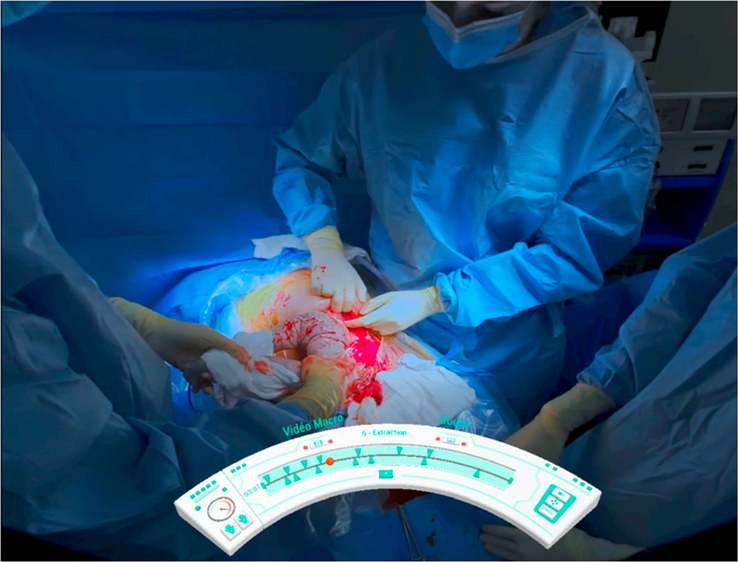
View from inside the HMD, showing the immersive video and an interface for navigating the content.

In the 3D immersive condition, participants wore a Meta Quest 2 HMD and hold a controller in each hand to interact with the interface. We developed custom software in unity to display the 3D video, along with a simple interface for navigating the content (Fig. 2). The video and audio content were identical for both groups.

In the 2D classic condition, the lecture was accessed through a web interface and displayed on a laptop screen. Participants wore a headset to listen to the audio.

### Procedure

**Figure 3:**
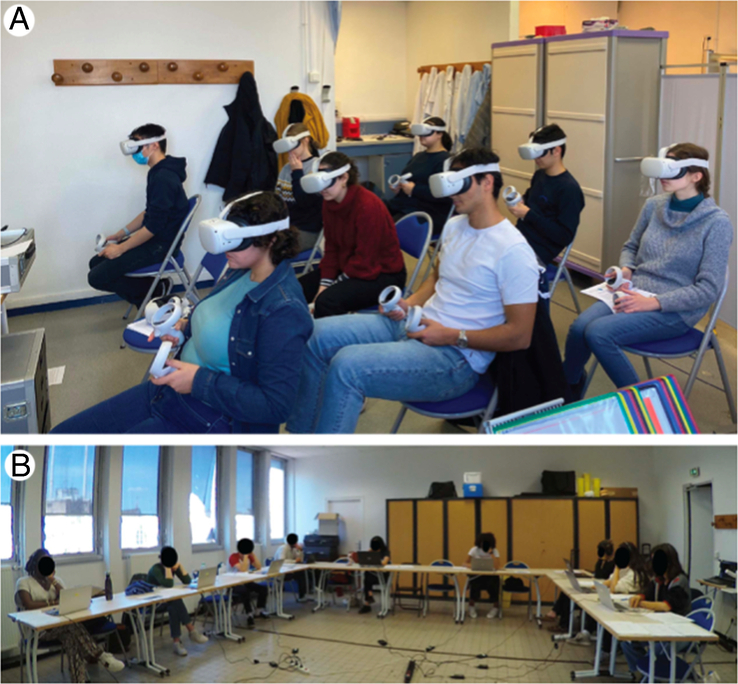
Setups. (A) Setup of the immersive group. (B) Setup of the classic group.

Participants were assigned an anonymous identifier and directed to one of the two classrooms corresponding to their experimental group (3D immersive or 2D surgical video) (Fig. 3). First, all participants completed a previewing questionnaire, then had 45 min to watch the C-section lecture at their own pace. Once finished, they completed a postviewing questionnaire and provided oral feedback if desired. One month later, participants received an e-mail asking them to complete a follow-up questionnaire using their anonymous identifier by following a web link.

### Questionnaires

All questionnaires were self-administered and are available in the appendix section. They were hosted on a private installation of the LimeSurvey platform. All questions used a 5-point Likert scale according to recommendation on this subject, ranging from ‘strongly disagree’^1^ to ‘strongly agree’^5^.

We created a set of 20 multiple choice questions (MCQs) to evaluate knowledge from the lecture. For each question, only one answer is correct out of five options. We followed the content validity method^18^. First, we developed the questions with two obstetrician-gynecologists based on the lecture content. Then, we emailed 10 experts in obstetrics and gynecology, inviting them to assess the relevance of our inquiries and suggestions. Each expert assigned a rating of either ‘relevant’ or ‘irrelevant’ to each question and provided open comments. We calculated a score (the average rating of the 20 questions) for each expert (ranging from 0 to 1) and computed the overall content validity score by averaging the 10 scores. The prevailing consensus was that most questions held relevance, resulting in an excellent content validity score of 0.85±0.38. Consequently, no questions were removed; however, we made minor adjustments to the phrasing of a few based on the experts’ feedback.

The previewing questionnaire consisted of two parts and is available in Appendix A (Supplemental Digital Content 1, http://links.lww.com/JS9/D579). It included questions about (i) sex, (ii) age, (iii) prior virtual reality experience, and (iv) prior participation in C-sections before the class (Demographic).

The first set of 10 multiple choice questions (MCQs) was part of the 20 questions designed for the study, assessing initial C-section knowledge.

The postviewing questionnaire consisted of four parts and was available in Appendix B (Supplemental Digital Content 1, http://links.lww.com/JS9/D579). It included the first set of 10 MCQs and the last 10 MCQs from the 20 questions designed for the study, assessing C-section knowledge. Our rationale for this format was to avoid bias from students focusing excessively on a specific set of questions, allowing for a thorough evaluation of knowledge retention. Questions are presented in the same order to each participant, but the sequence of questions is not structured (mixed) from Q1 to Q20. Students had no prior information about the questions they would be asked after watching the video lecture.

Three 5-point Likert scale questions for students to self-assess their readiness for their participating in an upcoming C-section with a responsibility of (i) an early medical student, (ii) resident, or (iii) junior assisted surgeon (Expected preparedness).

Four 5-point Likert scale questions for participants to note their engagement with the lecture content: (i) satisfaction, (ii) stimulation, (iii) involvement, and (iv) sensation of correct identify structures in the environment (Content engagement)^19^.

First, seven 5-point Likert scale questions asked participants to rate the tool’s usability, pedagogical value, and interest. These included: (i) control, (ii) ease of use, (iii) boost in self-confidence, and (iv) contribution to learning for future anatomy lectures.

Second, three questions investigated how participants interacted with the viewing tool, including (v) the amount of interface interactions (e.g. play/pause, change chapter, or skip a section), (vi) which parts of the video were watched, and (vii) any side effects encountered (Tool engagement).

The 1-month follow-up questionnaire consisted of two parts and is available in Appendix C (Supplemental Digital Content 1, http://links.lww.com/JS9/D579). Participants were asked to self-evaluate their involvement in the first C-section they observed during the month following the class, using four questions: (i) level of readiness, (ii) confidence, (iii) comfort, and (iv) feeling overwhelmed by information. Additionally, they specified (v) the time elapsed between the class and the C-section (1, 2, 3, or 4 weeks) and (vi) the context (urgent or scheduled) (First C-section experience).

Participants were also asked to self-evaluate their involvement in their first participation in a surgical procedure other than a C-section using the same questions as for the first C-section, except for (iv) feeling overwhelmed by information and (vi) the context (urgent or scheduled) (First Other Surgery Experience).

### Ethics

An ethics committee approval has been obtained for the study under the number CER - 2023 – REVAP. This work has been reported in line with Consolidated Standards of Reporting Trials (CONSORT, Supplemental Digital Content 2, http://links.lww.com/JS9/D580) Guidelines^20^.

### Statistical analysis

Statistical analyses were performed using R (Version 4.2.1). Descriptive analysis of quantitative data (age, initial C-section knowledge, and prior participation) was conducted according to the video group using the Fisher test. The Knowledge Retention score was analyzed according to the video group using a Student’s *t*-test. Expected preparedness (postviewing questionnaire B) and 1 month later preparedness (follow-up questionnaire) during the first C-section experience were analyzed according to the video group using the Fisher test. The tool engagement questions were quantitative and analyzed according to the video group using a Student’s *t*-test. For the knowledge retention score, we tested for confounding factors using a Pearson test on quantitative characteristics and a student’s *t*-test on binary characteristics. For Likert scale data, we tested for confounding factors using the Kruskal–Wallis test on quantitative characteristics and a Fisher test on binary characteristics. If we found a *P*<0.2, we adjusted the analysis by including the confounding factors.

## Result

### Participants characteristics

Two hundred sixty-nine fourth-year medical students were eligible for the study. Among them, 38 were excluded due to incomplete or missing answers to the knowledge retention questions. A total of 231 students were randomly assigned to either the immersive group (*n*=118) or the classic group (*n*=113).

The two groups were comparable, with no significant differences observed between the 3D immersive and 2D surgical video groups regarding gender, prior VR experience, or prior participation in C-sections. Additionally, no differences were noted in initial C-section knowledge between the two groups (Table 1). We have compared the prior participation to C-sections and initial C-section knowledge (pretest questionnaire). We see a strong correlation between the two, a Pearson test yields a *P*=0.00048 and R=.023. This means that the higher the number of prior observed C-sections, the higher the score of the initial questionnaire.

**Table 1:** Characteristics of participants according to video group.

Participant characteristics	Immersive group *N*=118 (%)	Classic group *N*=113 (%)	*P*
Classes			
December	40 (34%)	38 (34%)	0.97
February	40 (34%)	40 (35%)	
May	38 (32%)	35 (31%)	
Sex			
Female	80 (68%)	78 (69%)	0.72
Male	38 (32%)	35 (31%)	
Age, years (Mean +/− SD)	21.78 +/− 2.27	21.95 +/− 2.20	0.57
Prior virtual reality experience	45 (38%)	54 (48%)	0.14
Prior participation to C-sections			
0	92 (78%)	80 (71%)	0.38
1	12 (10%)	20 (17.5%)	
2	7 (6%)	10 (9%)	
3	4 (3.5%)	2 (2.5%)	
4	3 (2.5%)	0	
Initial C-section knowledge (Mean +/− SD)	6.46 +/− 1.81	6.88 +/− 1.81	0.08
Participation	118 (100%)	113 (100%)	

### Knowledge retention

**Figure 4:**
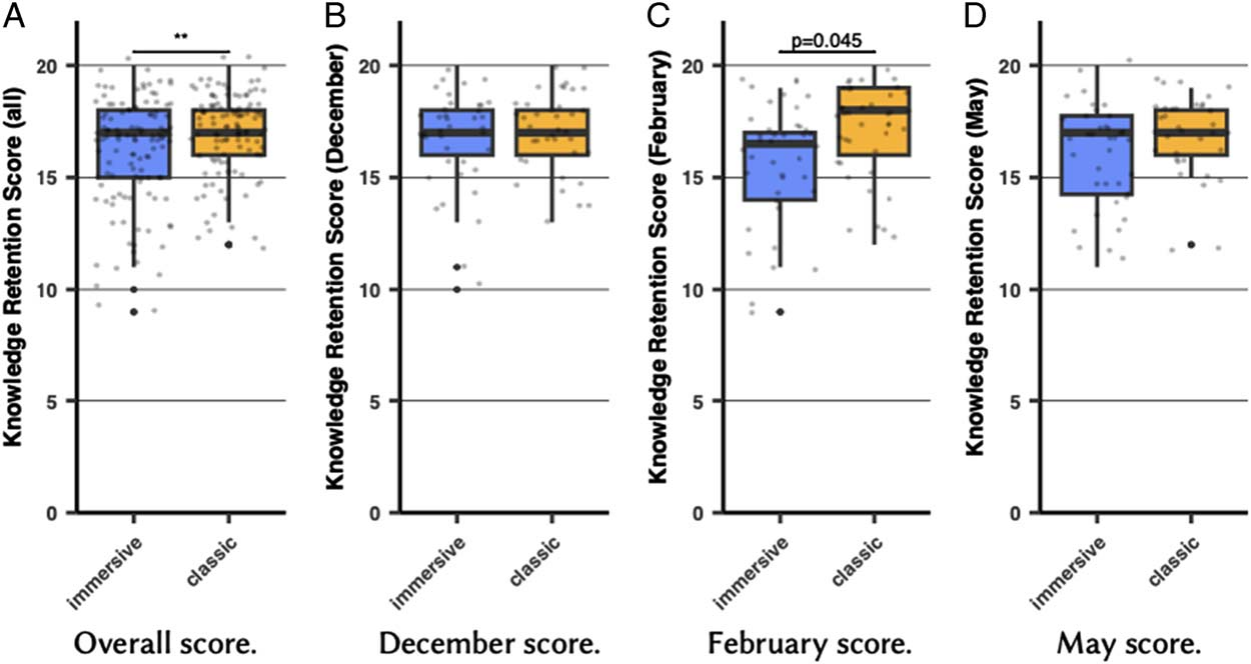
Box plots showing knowledge retention score after adjusting for initial C-section knowledge score and prior VR experience (**: *P*<0.01).

**Figure 5:**
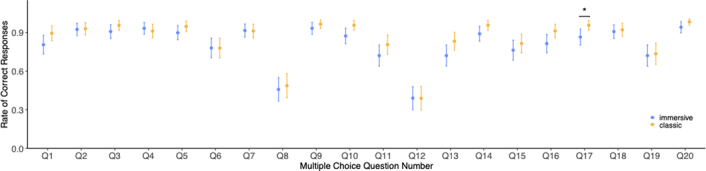
Plot showing knowledge retention scores by question. Bars are of 95% CI. (*: *P*<0.05).

All students completed the knowledge retention questionnaire (100%). After adjusting for initial C-section knowledge score and prior VR experience, the immersive video group showed a significantly lower knowledge retention score than the classic video group (mean=16.15 +/− 2.41 vs 17.04 +/− 1.83, t=−2.688, df=227, *P*<0.01) (Fig. 4). Each question was analyzed to investigate the reason behind these differences, but no differences were found, there were a difference in one question only (Fig. 5).

### Expected preparedness

Two participants did not reply to the expected preparedness questions. After adjusting for the initial C-section knowledge score and prior VR experience, we do not observe differences in expected preparedness after watching the video for the early medical student (t= 0.70, df=225, *P*=0.48), and there was a better-expected preparedness as a resident (t= 2.366, df=225, *P*=0.019).

### Content engagement

All participants answered this questionnaire. After adjusting for the initial C-section knowledge score and prior VR experience, we observe a significant difference in favor of the 3D immersive video group for all the content engagement questions: ‘I am satisfied to have participated to this virtual C-section session’ (t=2.152, df=223, *P*=0.033); ‘I felt stimulated by the tool’ (t= 4.687, df=224, *P*<0.001); ‘I felt involved in the experience’ (t=4.161, df=224, *P*<0.001); ‘I could correctly identify structures in the environment’ (t=3.355, df=224, *P*<0.001) (Annex 1, Supplemental Digital Content 3, http://links.lww.com/JS9/D581).

Results regarding tool engagement are presented in Annex 2 (Supplemental Digital Content 4, http://links.lww.com/JS9/D582) and 3 (Supplemental Digital Content 5, http://links.lww.com/JS9/D583).

### Preparedness after 1 month

After 1 month, 64 participants that had participated in a C-section responded to the follow-up questionnaire (26 in the immersive group and 38 in the classic group). Participants in the 3D immersive video group reported having felt more ready (t=2.48, df=62, *P*=0.016), self-confident (t=2.38, df=62, *P*=0.020), at ease (t=2.325, df=62, *P*=0.023), and less overwhelmed (t=−3.419, df=62, *P*<0.01) than those in the 2D video group (Annex 4, Supplemental Digital Content 6, http://links.lww.com/JS9/D584).

### Side effects

Participants in the 3D immersive video condition reported more adverse effects (t=9.56, df=223, *P*<0.001) than those using classic video (mean=1.99±1.47 vs. 0.47±0.72). In the 3D immersive video group, 32.7% of participants reported visual strain, 28.6% experienced heaviness in the head, 24.5% reported headaches, 8.2% noted general fatigue, 6.1% experienced dizziness, 6.1% reported nausea, 2% reported other symptoms, and 26.5% reported no side effects.

## Discussion

Our results show that viewing a 3D C-section video through an HMD, compared to viewing a 2D C-section video on a conventional screen, increased the sense of stimulation, satisfaction, involvement, and the perception of having better identified anatomical structures. It gives the students the feeling of being well-prepared for a C-section procedure and arouses the curiosity of students who expressed their desire to use immersive videos for other courses. Notably, immersion enhanced their feeling of being prepared for real-life surgery as a resident, and when reflecting on their first C-section, the 3D immersive video group reported feeling more prepared than the 2D video group. Our findings contribute to the understanding of immersive visualization technologies, providing evidence that aligns with the literature, which suggests that the impacts of immersion are complex.

While immersive VR environments are widely used in other fields, their application in medical education is not yet widespread due to a lack of research^21^. In mathematics, Parong and Mayer formulated two contrasting hypotheses regarding immersive VR environments^22^. The immersion-as-distractor hypothesis, supported by the Cognitive Load Theory and the Cognitive Theory of Multimedia Learning, suggests that the perceptual richness of immersion increases cognitive load, thereby decreasing the resources available for learning^22,23^. Conversely, the immersion-as-motivator hypothesis, supported by the Cognitive Affective Theory of Learning with Media (CATLM), posits that the enhanced affective processing from immersion leads to increased cognitive processing, resulting in greater cognitive effort dedicated to understanding the content^21^. In our course, it appears that the surgical domain does not significantly differ from learning mathematics. Our data indicate that immersion may serve as a distractor, as evidenced by lower knowledge retention scores in the immersive group compared to the classic group. Additionally, studies have identified various challenges to learning in surgical settings, including the physically demanding environment, the high emotional impact, the dual-purpose task of performing and teaching, and the complex social relationships that need to be managed. These factors can lead to unclear learning objectives, as well as feelings of fear, anxiety, humiliation, and intimidation^24,25^. Although learning may not improve immediately, immersion can create conditions that facilitate learning during subsequent visits to the OR. Visualizing video in immersion can reduce barriers to knowledge absorption, as students feel ‘transported’ into the room and experience the procedure from a realistic and embodied perspective.

Our study demonstrated an improvement in both prospective and retrospective preparedness. This provides new evidence suggesting that immersive experiences enhance situated learning by allowing students to engage directly in the action. The OR is a setting where knowledge is transmitted through observation and participation in situated actions. Arriving prepared to the OR has been identified as a crucial factor in overcoming these obstacles^26^. Traditional simulations of the OR environment, such as using mannequins or role-playing, have been shown to reduce anxiety and subsequently enhance student preparedness^27^. We hereby highlight that immersive visualization technologies can also effectively prepare students by providing a clear understanding of what they will encounter. We focused on surgical steps addresses one of the often-overlooked areas students to prepare for, as they strongly prepares for C-section^28^. The integration of artificial intelligence (AI) with immersive video technology indeed holds great potential for advancing medical education. AI tools like ChatGPT (Generative Pretrained Transformer) can enhance learning experiences by offering detailed, accurate, and relevant anatomical information tailored to students’ needs^29^.

In our study, the immersive 3D video group demonstrated significant content engagement, feeling stimulated and more involved with this tool. Students who are genuinely interested in a subject experience a marked increase in their learning potential. The enthusiasm generated by captivating subjects can transform the learning process into a rewarding and motivating experience. When students are emotionally invested in their studies, they become more receptive to information and more inclined to assimilate knowledge for the long term. This connection between interest and learning is rooted in the principle of cognitive engagement, where curiosity and passion drive a more effective knowledge-acquisition process^30^. This is evidenced by the fact that students in the immersive group expressed a desire for more courses using the proposed video tool.

It is important to note that most participants in our study were not experienced users of immersive environments, and our results may, therefore, be influenced by a novelty effect that could act as a distractor. Further research is needed to identify the factors that either hinder or enhance the potential of immersion, such as the application domain or participant characteristics. By creating a stimulating teaching approach, educators can guide students toward intellectual fulfillment and academic success.

Our randomized study included a large population of students from a real medical school. However, our method has some limitations and potential biases. We chose to use an HMD that displays 3D 180° video to operationalize an immersive content delivery system. Other systems should also be studied, such as screens with 3D glasses. Additionally, future research could compare HMD 3D video with 3D video on screens and 2D screens to isolate the effects of 3D video and immersion. Another limitation is the potential for distraction; future studies should include a group that can wear the device for as long as they wish until the novelty wears off. It would also be valuable to compare our learning tool to actual OR experiences to assess the impact of cognitive load on learning these procedural steps. Furthermore, this study focuses on students’ perceptions rather than evaluating clinical practice after being exposed to immersive 3D video. The immersive video group perceived that they were better able to identify anatomical structures; however, we did not assess whether the anatomical structures identified by the students were correct. Also, while we assessed participants’ impressions through a questionnaire 1 month later, we did not conduct a clinical evaluation to understand the impact on patient care.

Regarding potential biases, we conducted a randomized study to minimize differences between the two groups. However, we cannot entirely rule out variations in the students’ levels, even though we attempted to match them. Additionally, since this was not a blind study, participants’ awareness of being in the immersive video group may have influenced their use of the tool. A further bias at the 1-month mark could arise from the acquisition of knowledge of each student, and self-selection bias may have occurred since not all students responded to the follow-up questionnaire.

Our study demonstrated that the immersive video method is feasible in a university setting. This approach allows a larger number of students to access the OR, unlike the one or two students who typically participate over several weeks in a real OR, where the number of simultaneous learners is limited. Additionally, it reduces the number of instructors required and the time spent on real patients to achieve a certain level of competency. Ideally, 3D cameras should be widely implemented in the OR to film procedures and create continuous video resources for students. To better target the use of these systems, we identify two key research directions. First, studying the characteristics of individuals who are more responsive to immersive technologies, particularly in environments where there are more students than available resources. For example, exploring the impact of visual-spatial abilities could help determine which students would benefit most from accessing the immersive system. Second, it is important to understand which types of content are best suited for immersive experiences. For instance, stereoscopic displays without HMDs can enhance the understanding of immersion^31^. This understanding can guide decision-makers on which content should be delivered in immersive formats to maximize benefits.

## Conclusion

Our results showed that 3D surgical immersive video vs. 2D surgical video, enhances the sense of stimulation, satisfaction, involvement, and the perception of having better identified anatomical structures. For early medical school students where access to the OR is limited, this tool appears to a significant step forward in surgical pedagogy. However, the precise understanding of its pedagogical value required further investigation and refinement.

## Ethical approval

Sorbonne Université, CNRS, INSERM, ISIR, Institut des Systèmes Intelligents et de Robotique, 75005 Paris, France CER - 2023 – REVAP.

## Consent

We confirm.

## Source of funding

No funding.

## Author contribution

A.F.: writing the paper and data analysis; E.J.: data collection and data analysis; R.L’.H.: data collection, data analysis, and study concept; P.G.: study concept; I.A. and G.C.: writing the paper, data analysis, and study concept.

## Conflicts of interest disclosure

The authors declare no conflicts of interest.

## Research registration unique identifying number (UIN)

CER - 2023 – REVAP.

## Guarantor

Amelia Favier, Ignacio Avellino, and Geoffroy Canlorbe.

## Data availability statement

Not publicly available. Available upon reasonable request.

## Provenance and peer review

Not commissioned, externally peer-reviewed.

## Supplementary Material

SUPPLEMENTARY MATERIAL
