# Supplementary figures and images for: Impacts of immersive 3D videos on students’ surgical learning compared to 2D videos: a randomized controlled trial
Source: Int J Surg. 2024 Nov 25;110(12):7832–9. doi: 10.1097/JS9.0000000000002146 (PMC11634195; doi:10.1097/JS9.0000000000002146)

Appendix : Questionnaires


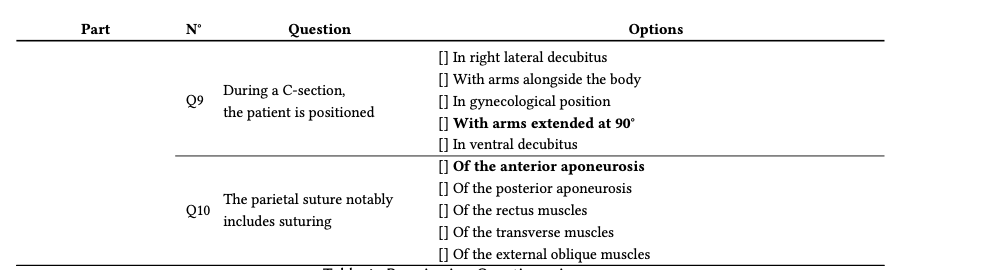

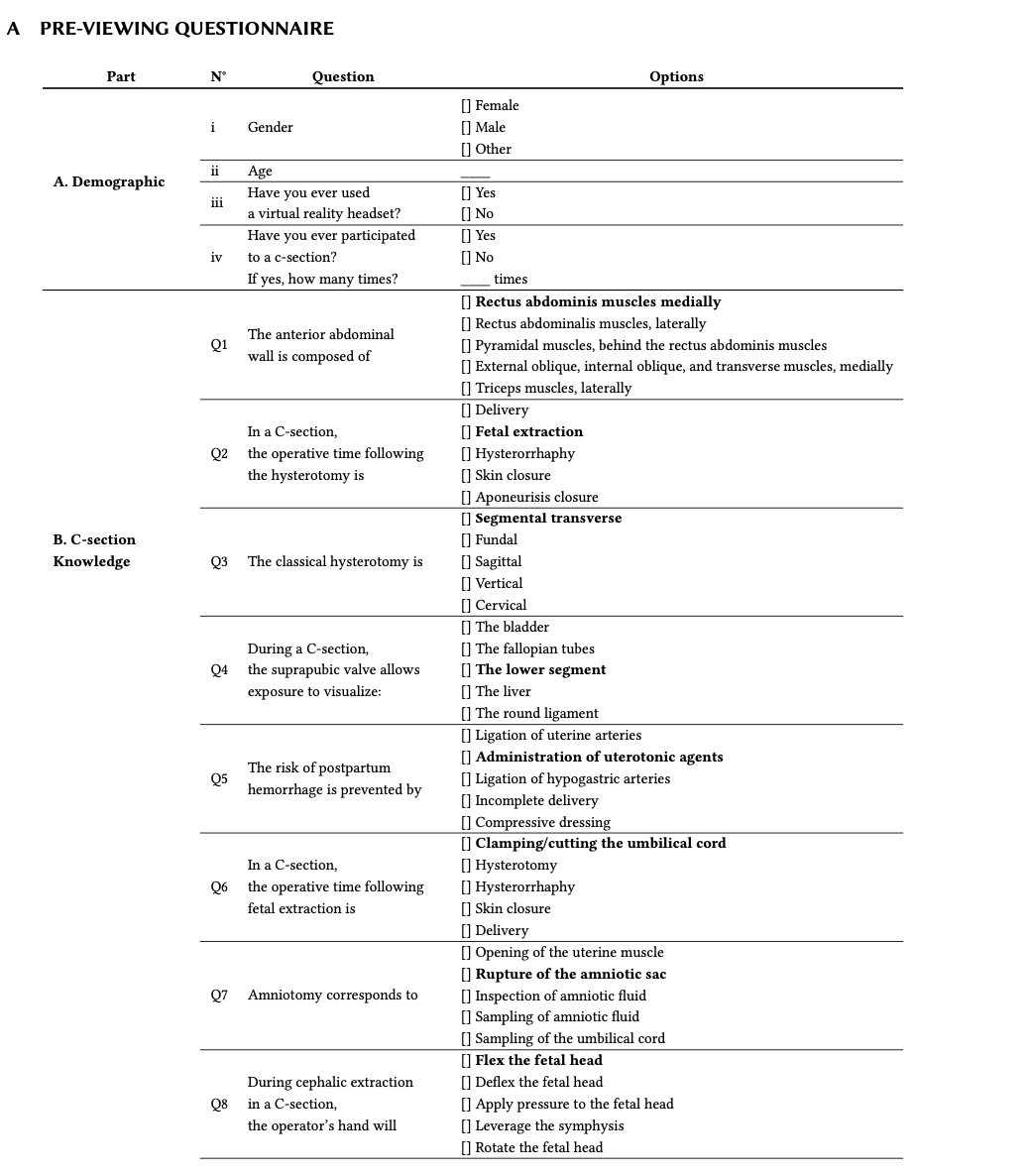


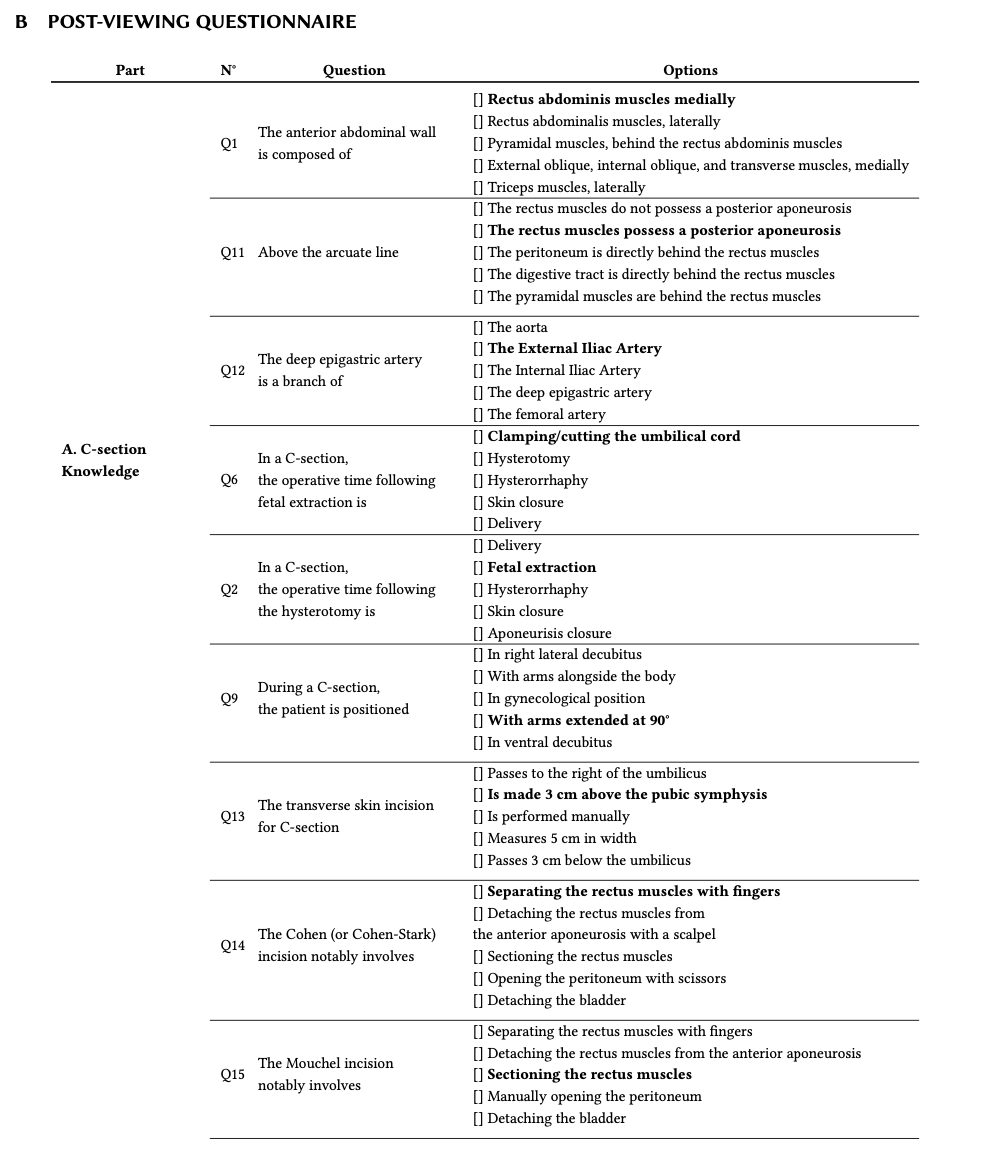


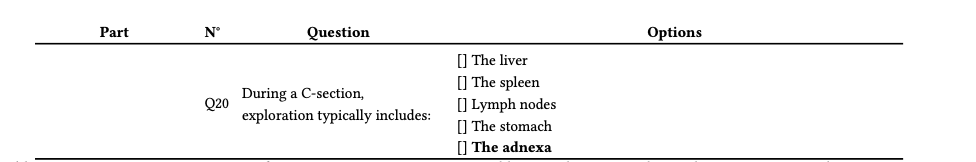

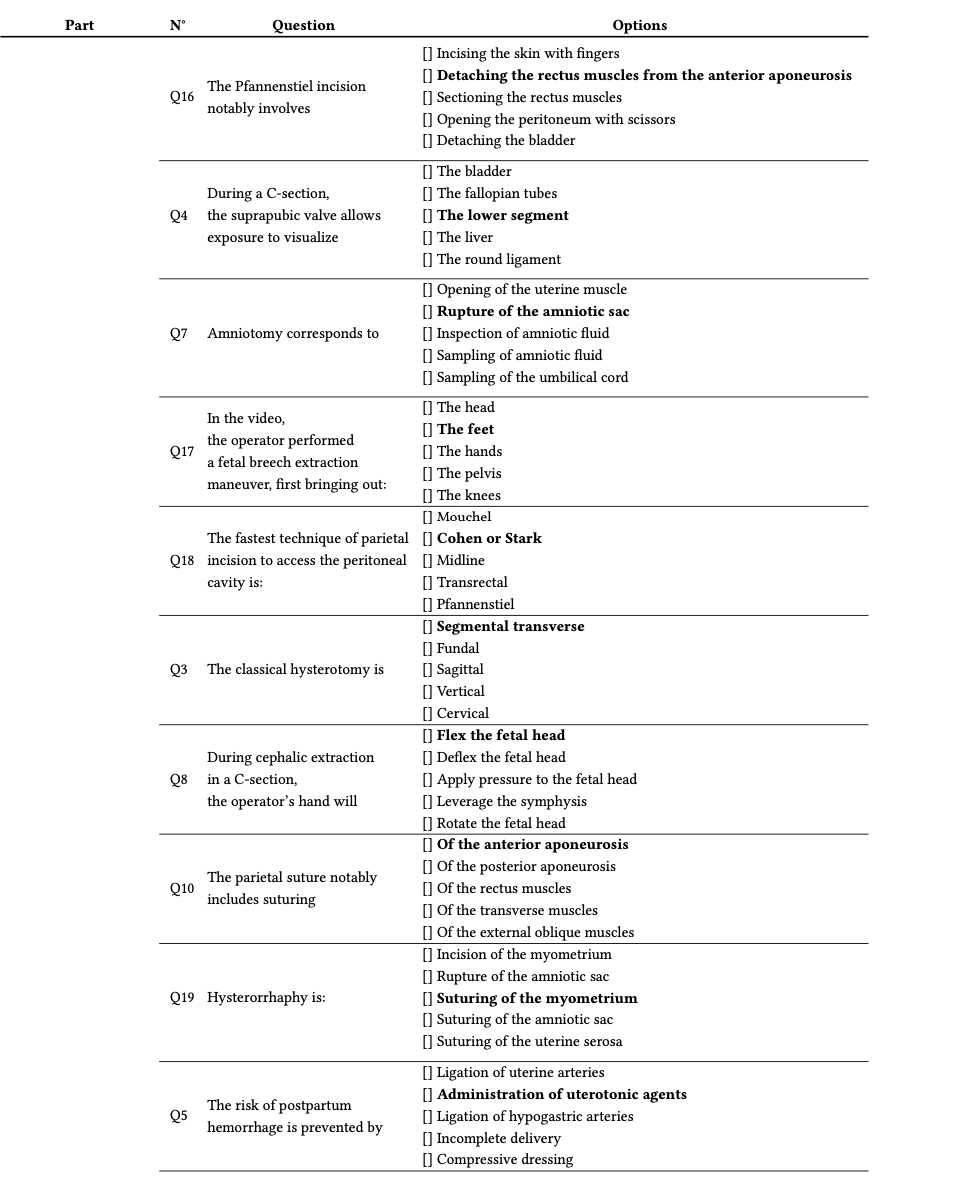


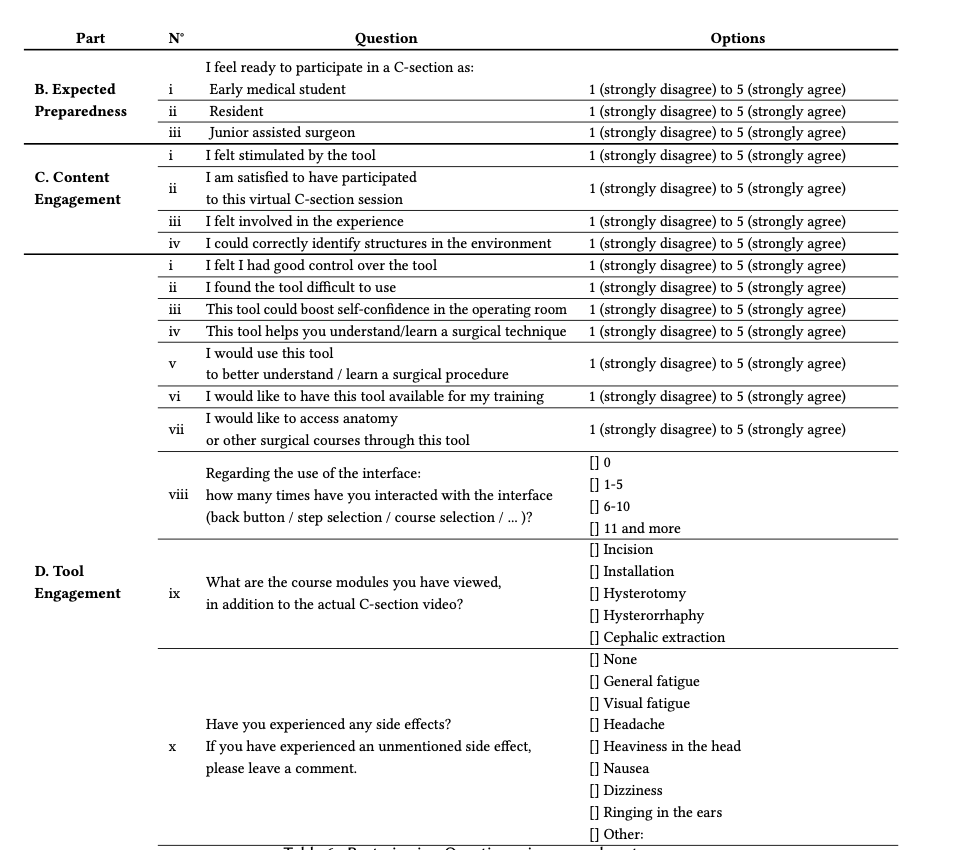


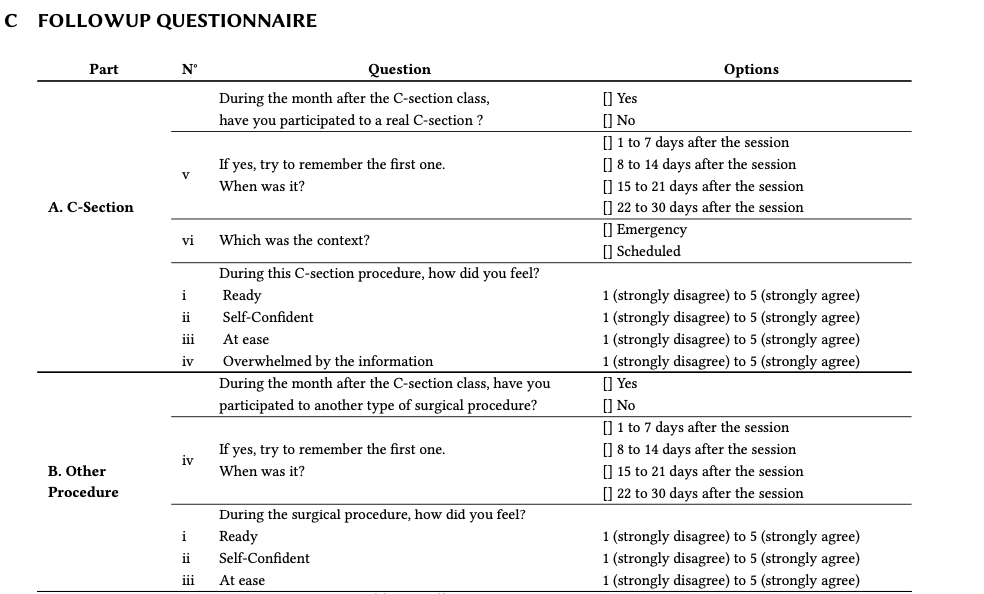

Supplement: SUPPLEMENTARY MATERIAL [file js9-110-7832-s001.docx]

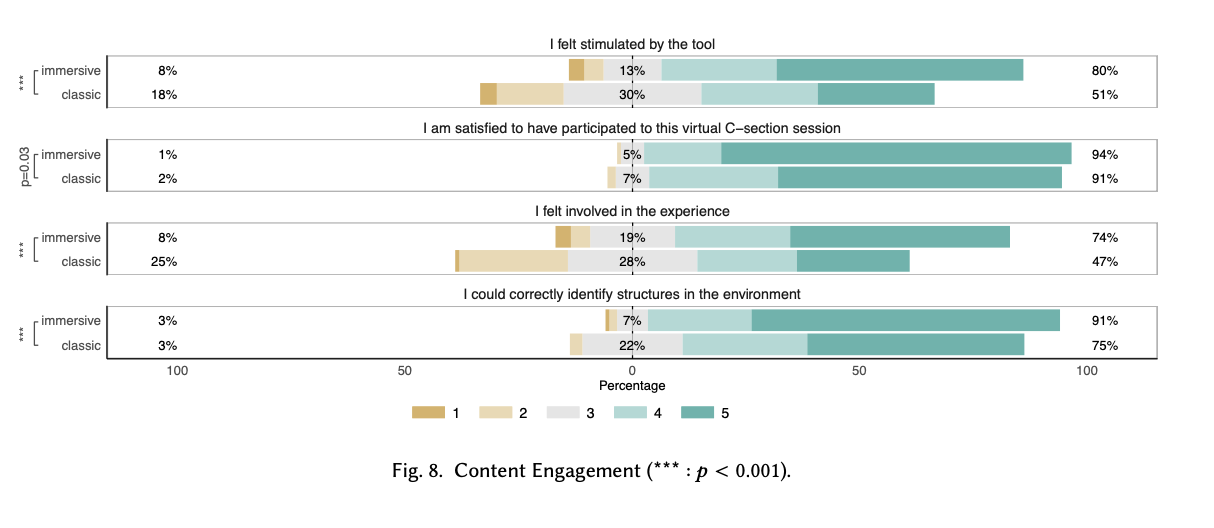


*** *** p=0.033 ***

Annex 1: Content engagement.

(***:p<0.001)

Supplement: SUPPLEMENTARY MATERIAL [file js9-110-7832-s003.docx]

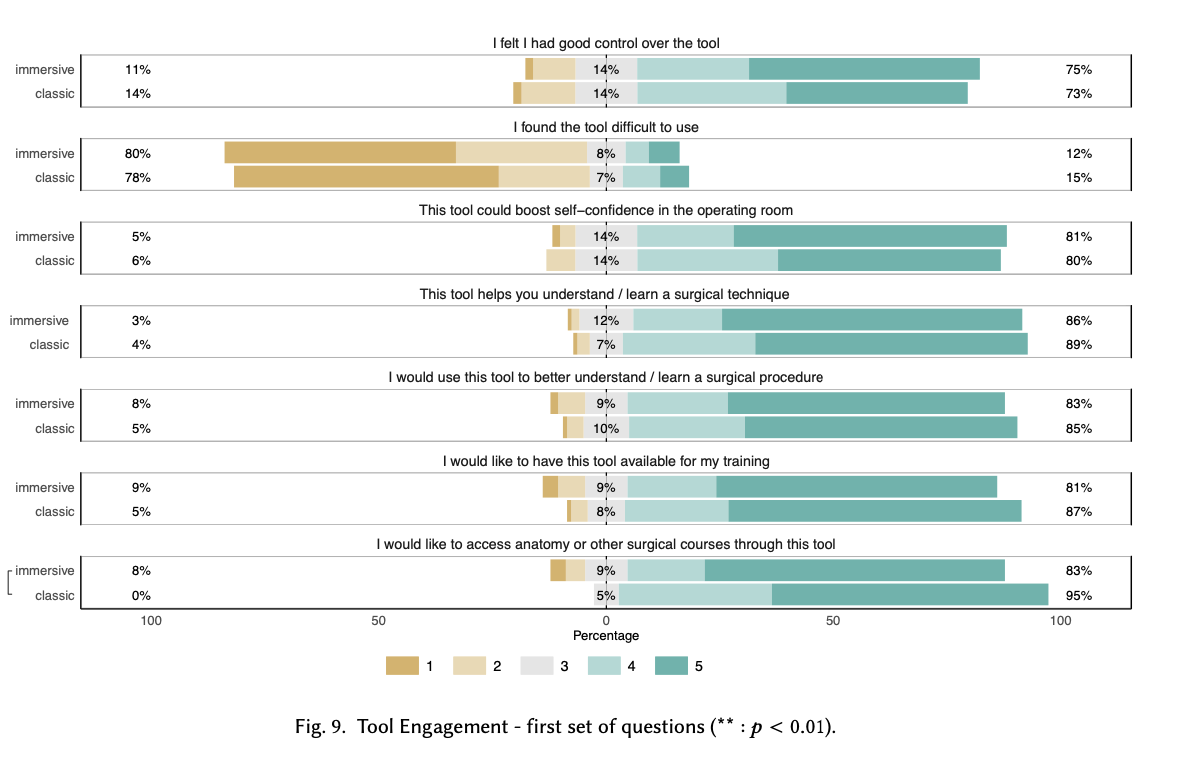


Annex 2 (Figure): Tool Engagement: First set of questions.

Supplement: SUPPLEMENTARY MATERIAL [file js9-110-7832-s004.docx]

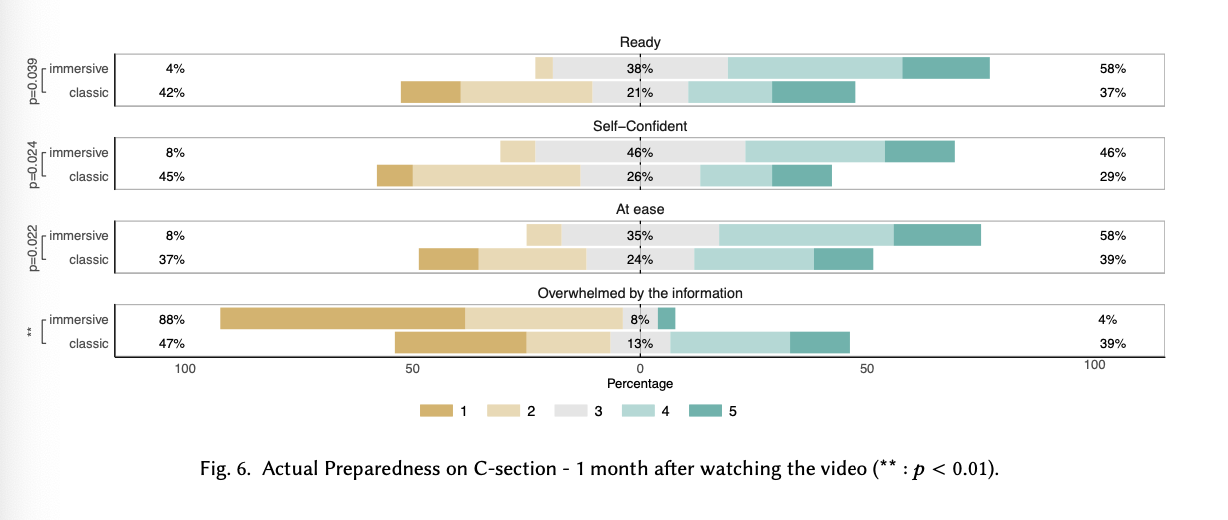


** p=0.023 p=0.020 p=0.016

Annex 4 : Preparedness after one month for a C-section. (**:p<0.01)

Supplement: SUPPLEMENTARY MATERIAL [file js9-110-7832-s006.docx]
