# Supplementary material for: Impacts of immersive 3D videos on students’ surgical learning compared to 2D videos: a randomized controlled trial
Source: Int J Surg. 2024 Nov 25;110(12):7832–9. doi: 10.1097/JS9.0000000000002146 (PMC11634195; doi:10.1097/JS9.0000000000002146)
Supplement: SUPPLEMENTARY MATERIAL [file js9-110-7832-s005.docx]

Annex 3 (Table): Tool Engagement: Second set of questions

| Question | Immersive Group  N=118 (%) | Classic Group  N=113 (%) | p |
| --- | --- | --- | --- |
| Number of interface interactions  0  1-5  6-10  >11  NA | 5 (4%)  63 (53%)  32 (27%)  18 (15%)  0 | 3 (2%)  52 (46%)  38 (34%)  16 (14%)  4 (3%) | 0.21 |
| Courses viewed in addition to the actual C-section video (mean +/- SD) | 4.89 +/- 0.50 | 4.69 +/- 0.95 | 0.033* |
| Side effects experiences (mean +/- SD) | 1.99 +/- 1.47 | 0.47 +/- 0.72 | <0.001*** |
